# HOTAIR: a potential metastatic, drug-resistant and prognostic regulator of breast cancer

**DOI:** 10.1186/s12943-023-01765-3

**Published:** 2023-03-30

**Authors:** Ganji Seeta Rama Raju, Eluri Pavitra, Sai Samyuktha Bandaru, Ganji Lakshmi Varaprasad, Ganji Purnachandra Nagaraju, Rama Rao Malla, Yun Suk Huh, Young-Kyu Han

**Affiliations:** 1 Department of Energy and Materials Engineering, Dongguk University–Seoul, Seoul, 04620 Republic of Korea; 2 NanoBio High-Tech Materials Research Center, Biological Sciences and Bioengineering, Inha University, Incheon, 22212 Republic of Korea; 3 Baton Rouge General Hospital, Baton Rouge, LA-70809 USA; 4 School of Medicine, Division of Hematology and Oncology, University of Alabama, Birmingham, AL 35233 USA; 5 Cancer Biology Laboratory, Department of Biochemistry and Bioinformatics, GIS, GITAM (Deemed to be University), Visakhapatnam, Andhra Pradesh 530045 India

**Keywords:** Breast cancer, Drug resistance, HOTAIR, Metastasis, Prognosis

## Abstract

HOX transcript antisense intergenic RNA (HOTAIR) is an oncogenic non-coding RNA whose expression is strongly correlated with the tumor grade and prognosis of a variety of carcinomas including breast cancer (BC). HOTAIR regulates various target genes via sponging and epigenetic mechanisms and controls various oncogenic cellular and signaling mechanisms including metastasis and drug resistance. In BC cells, HOTAIR expression is regulated by a variety of transcriptional and epigenetic mechanisms. In this review, we describe the regulatory mechanisms that govern HOTAIR expression during cancer development and explore how HOTAIR drives BC development, metastasis, and drug resistance. In the final section of this review, we focus on the role of HOTAIR in BC management, therapeutic treatment, and prognosis, highlighting its potential therapeutic applications.

## Introduction

HOX transcript antisense intergenic RNA (HOTAIR) is a novel class of oncogenic long non-coding RNA (lncRNA) that belongs to subclass intergenic lncRNA that tightly regulates genes associated with the embryonic development of mammals. The human HOTAIR gene is found between *HOXC11* and *HOXC12* genes on chromosome 12q13.13. A polyadenylated RNA, HOTAIR is transcribed from a non-coding strand of the HOXC gene cluster containing 2158 nucleotides and 6 exons via RNA polymerase II, and the nascent RNA transcript is spliced into five variants via alternative splicing. The transcript does not encode proteins but regulates the expression of distant target genes in a trans-regulatory manner.

HOTAIR modulates chromatin remodeling and epigenetic changes by acting as a scaffold for protein complexes that modify histones and by regulating the different states and dynamics of chromatin via binding with polycomb repressive complex 2 (PRC2), a specific complex that remodels chromatin. It is also known to coordinate chromatin-modifying enzymes and gene expression. It mediates regulation of gene silencing by promoting methylation of H3K27 and the demethylation of H3K4 via directing PRC2 and LSD1 complex to a specific site on the target genes [1]. HOTAIR embodies the second level of post-transcriptional regulation of HER2 expression by acting as competitive endogenous RNA to limit miR-331-3p [2].

HOTAIR is also known as an oncogene, with its expression strongly correlated with the grade and prognosis of a variety of cancers. In particular, it represses the functions of tumor suppressor genes by facilitating the recruitment of EZH2 for the catalysis of H3K27 triple-methylation and promotes tumorigenesis by regulating cellular and molecular mechanisms. HOTAIR is also associated with the epigenetic regulation [3], cancer susceptibility [4], poor survival [5], tumor recurrence [6], immune escape [7], and oncogenic signaling pathways [8]. It promotes tumor growth [9], cell cycle progression [10], cancer metastasis and drug resistance [11], radiation resistance [12], invasion and migration of cells [13], tumor induced angiogenesis [14], DNA damage response [15], inflammation [16], stemness [17], multidrug resistance [18], metastasis-promoting phenotypes [19], epithelial–mesenchymal transition (EMT) [20], while also and inhibiting apoptosis [21] by modulating miRNAs. Moreover, HOTAIR regulates the angiogenesis as well as permeability of blood-tumor barrier via controlling Wnt/b-catenin axis [22] and contributes to biogenesis and the secretion of tumor exosomes [23], the modulation of exosomal miRs [24], and the membrane fusion of exosomes by affecting the colocalization of VAMP3 and SNAP23 via the mTOR-signaling-dependent phosphorylation of SNAP3 [25]. Therefore, over the past decade, HOTAIR has been recognized as a critical regulator of mechanisms associated with breast cancer (BC) progression and understanding the cancer-facilitating mechanisms of HOTAIR could assist in developing novel treatment strategies for BC.

In the past decade, few reviews have summarized the oncogenic nature of HOTAIR [25–29]. As an advance over the existing literature, the present review provides an up-to-date and comprehensive overview of the regulatory mechanisms that govern HOTAIR expression during BC development, its role in driving BC metastasis and drug resistance, and its potential as a therapeutic target. This review also provides a more in-depth analysis, presents new and relevant information, and offers a unique perspective on the epigenetic and post-transcriptional mechanisms that regulate HOTAIR expression, including the role of microRNAs and RNA-binding proteins. It also highlights recent advances in the field, such as new findings on the function of HOTAIR in drug resistance and the potential of targeting of HOTAIR for BC treatment. This review also provides insights into the limitations of the current understanding of HOTAIR and offer a critical analysis of the existing literature, thus facilitating a better understanding of the potential clinical implications of HOTAIR in BC management.

## Mechanisms involved in the regulation of HOTAIR and its association with cancer

**Fig. 1:**
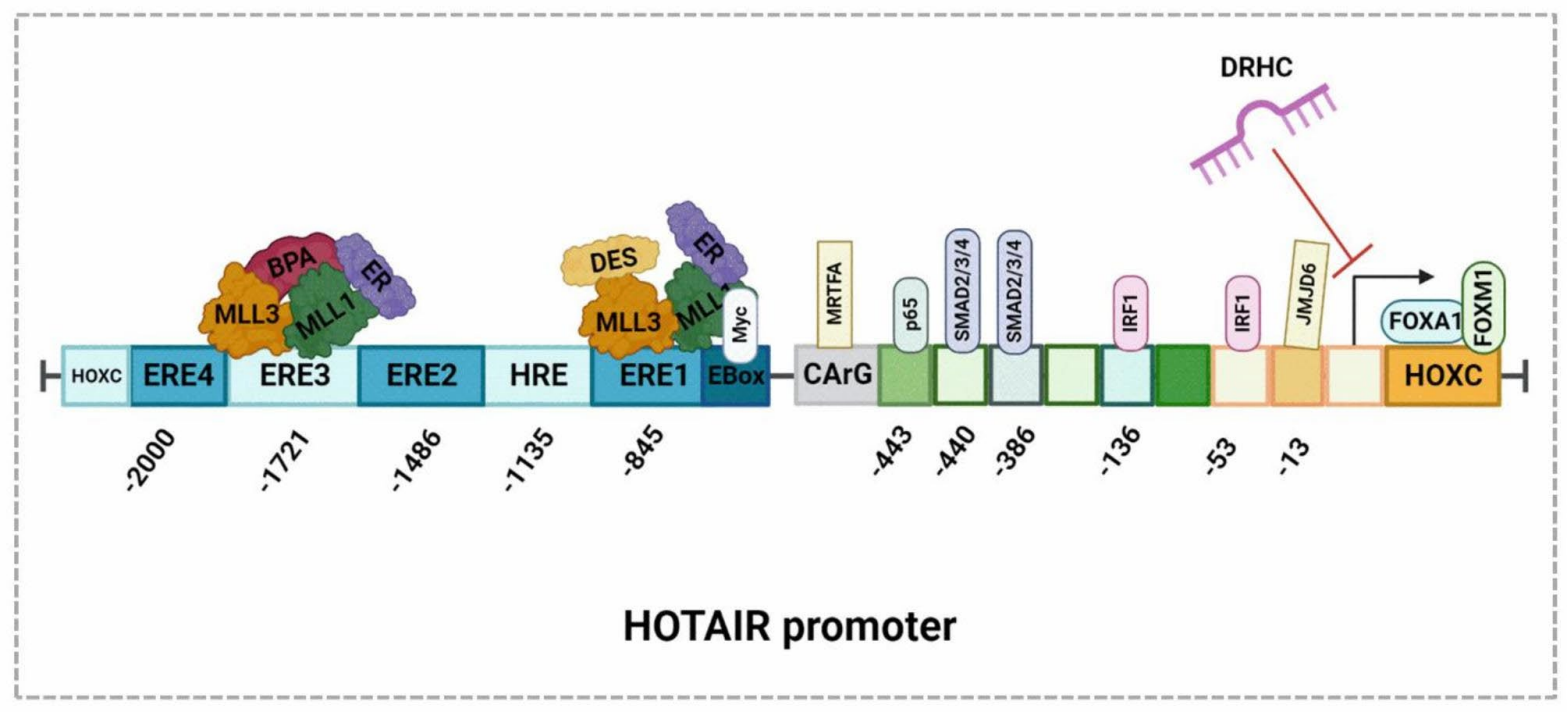
BisPhenol A, diethylstilbesterol, and IRF1 promote the overexpression of HOTAIR by binding to the corresponding gene promoter elements. Bisphenol A and diethylstilbesterol independently interact with estrogen receptors in the cytoplasm, translocate into the nucleus, associate with the methyl transferases MLL1 and MLL3, bind to the estrogen receptor elements ERE1 and ERE3, and then activate the overexpression of HOTAIR. IRF1 binds to the downstream sequence in between 53–64 and 136–148 within the promoter region and activates the overexpression of HOTAIR. P65, which is an important component of the NF-kB signaling pathway, directly binds to the sequence between − 443 and − 475 within the promoter element and regulates the expression of HOTAIR. Myocardin Related Transcription Factor(MRTF-A) binds to the CArG box located in the HOTAIR promoter and facilitates the association between SRF1 and RNA POL II, which is involved in the expression of HOTAIR. Transcription factor c-Myc binds specifically to the E-box sequence within the HOTAIR promoter region and increases its expression. FOXA1 and FOXM1 interact with the HOXC locus positioned adjacent to the HOTAIR gene promoter and regulates its expression LncRNA DRHC suppresses HOTAIR expression and inhibits the proliferation of triple negative BC cells. SMAD2/3/4 interacts with the promoter region of HOTAIR between − 386 and − 398 and between − 440 and − 452 to facilitate the transcription of HOTAIR, while MJD6 induces the expression of HOTAIR by directly binding to its promoter in the − 13 to −103 region

HOTAIR is a master regulator of metastasis that itself regulated by a number of transcriptional and epigenetic mechanisms. Indeed, over the past decade, research has identified a diverse range of regulatory mechanisms that modulate HOTAIR expression via promoter interaction in BC (Fig. 1). In particular, the promoter regions of HOTAIR contain an interaction site for AP1 and Sp1 transcription factors and binding sites for estrogen response elements (EREs) and hypoxia response elements (HREs). For example, Bisphenol-A, a toxic chemical that affects the nervous, reproductive, and immune systems, transcriptionally upregulates HOTAIR via the interaction with an ERE. Diethylstilbesterol, a synthetic form of estrogen, also induces the transcription of HOTAIR by interacting with an ERE [30].

Tumor suppressor proteins are also known to regulate HOTAIR expression. For example, interferon regulatory factor 1 (IRF1) regulates HOTAIR expression by binding at the 53–64 and 136–148 positions of the promoter region, which contains an interferon-stimulated response element (ISRE) [31]. Once IRF1 binds to the promoter region of HOTAIR, it recruits co-repressors such as histone deacetylases (HDACs) and other chromatin-modifying proteins that modify the chromatin structure, leading to a repressive chromatin environment that prevents the transcription of HOTAIR and thus its expression. This prevents the downstream effects of HOTAIR on gene regulation, chromatin remodeling, and cancer progression. In addition, the transcription levels of HOTAIR are altered by methylation of the intergenic CpG island found between the HOTAIR and HOXC12 genes. The highly ordered structure of HOTAIR confers on it a scaffolding function. It consists of four interdependent folded domains, two of which are functionally involved in binding transcription factors through transcription binding sites, one at the 5’end and another one at the 3’ end [32].

MRTF-A and SRF can also affect the expression of HOTAIR. Overexpression of MRTF-A enhances the expression of HOTAIR by physically interacting with the CArG box in the HOTAIR promoter site (Fig. 1) and facilitating the SRF-dependent association of RNA Pol II [33]. In BC, key components of ER signaling are forkhead-Box transcription factors (FOXA1 and FOXM1), which regulate the transcription of HOTAIR via interacting with the HOXC locus (Fig. 1). Downregulation of FOX proteins using specific siRNAs suppressed the expression of HOTAIR, suggesting FOX protein mediated regulation of HOTAIR [34].

The transcription factor c-Myc also regulates HOTAIR in BC cells by binding to the gene at a specific E-box sequence in the promoter regions of the HOTAIR gene [35]. After c-Myc binds to the HOTAIR promoter, it recruits histone acetyltransferases to the HOTAIR promoter that modify the chromatin structure by adding acetyl groups to histone proteins, which leads to the formation of an open chromatin environment that allows for gene transcription and results in greater HOTAIR expression. This allows for the downstream effects of HOTAIR on gene regulation, chromatin remodeling, and cancer progression. Another study by Li et al. [36] demonstrated that HOTAIR acts as a scaffold for c-Myc, thus influencing the upregulation of cyclin A, eIF4E, and LDHA in BC cells. Mechanistically, HOTAIR brings c-Myc and other proteins close to the promoter regions of cyclin A, eIF4E, and LDHA, which leads to the formation of a transcriptional activation complex that results in the upregulation of these three proteins. Their study also demonstrated that HBXIP facilitates the recruitment of HOTAIR and histone demethylase to the promoter regions of target genes by directly interacting with the leucine zipper motifs of c-Myc. SMAD2/3/4 interacts with the promoter region of HOTAIR between − 386 and − 398 and between − 440 and − 452 to facilitate the transcription of HOTAIR, MJD6 induces the expression of HOTAIR by directly binding to its promoter in the − 13 to −103 region.

The transcription of HOTAIR is also enhanced by IL-6/STAT3 signaling [37]. Transcriptionally, HOTAIR is regulated by RhoC-ROCK signaling in BC. The disruption of this pathway using chemical inhibitors or specific siRNAs downregulates the expression of HOTAIR. In addition, a bioinformatics-based transcription factor prediction study by Zhang et al. [38] identified p65, a component of NF-kB signaling pathways on the promoter region of HOTAIR from − 475 to − 443 bp. Chromatin precipitation further confirmed the binding of phosphorylated p65 to the HOTAIR promoter. In addition, the silencing of p65 using siRNA reduces the expression of HOTAIR by negatively regulating TNF-α-induced protein 8-like 2.

In BC, the transcription of HOTAIR is induced by estradiol (E2) via the interaction with the estrogen receptor. E2 facilitates the binding of its receptors and coregulators to the promoter regions of the HOTAIR promoter and enhances the recruitment of RNA polymerase II by increasing histone acetylation and histone H3 lysine-4 trimethylation [39]. In addition, E2 promotes HOTAIR expression in hormone receptor-negative BC via estrogen-receptor-independent mechanisms by negatively regulating miR-148a via G-protein-coupled estrogen receptor-1 signaling [40]. In Claudin-low BC cells, a novel isoform of HOTAIR is induced by the extracellular matrix via activation of α2 integrin/SRC kinase signaling, thus promoting invasive growth [41]. The promoter of this isoform exhibited enhanced histone H3 lysine 4 trimethylation and BRD4 binding. However, the silencing of BRD4 significantly reduces HOTAIR expression. This study indicates that BRD4 promotes the transcription of HOTAIR by binding with the histone markers of the promoter.

HOTAIR is also regulated by upstream mediators. In addition to the direct regulation of target gene expression, HOTAIR is transcriptionally regulated by heteronuclear ribonucleoproteins (hnRNP A2/B1) via RNA–RNA interaction. Furthermore, the silencing of A2/B1 decreases the HOTAIR-mediated invasion and migration of BC cells [42]. Another study by Wang et al. [43] reported that the protein-coding gene EGFR positively controls the expression of HOTAIR in TNBC cells. This study demonstrated that an aptamer coupled with siRNA against HOTAIR significantly reduces the growth and metastasis of triple-negative BC (TNBC) cells by regulating the expression of HOTAIR. Biswas et al. [44] conducted a microarray analysis of TNBC cells and found that the upregulation of HOTAIR is mediated by Jumonji domain-containing 6 (JMJD6). This study observed a linear correlation between the JMJD6 and HOTAIR levels in BC tissues. Furthermore, chromatin precipitation assay found that JMJD6 induces the expression of HOTAIR by directly binding to its promoter in the − 13 to − 103 region.

In BC cells, BRCA1 is reported to negatively upregulate HOTAIR expression by limiting the interaction of EZH2 with the HOTAIR promoter, while the inhibition of BRCA1 increases the invasion and migration of BC cells via HOTAIR [45]. In addition, lncRNA downregulated in hepatocellular carcinoma (DRHC), which is a key tumor-suppressor IncRNA in TNBC, negatively regulates the expression of HOTAIR and inhibits the proliferation of TNBC cells. However, forced HOTAIR expression mitigates the effect of DRHC on proliferation, suggesting that DRHC is an upstream regulator of the HOTAIR-dependent proliferation of TNBC cells [46].

Recently, a study by Porman et al. [47] found that the m6A modification of HOTAIR, a long noncoding RNA, is critical for the proliferation and invasion of TNBC cells. The methylated site A783 interacts with the m6A reader YTHDC1, promoting the gene repression and chromatin association of HOTAIR, while the loss of this modification leads to the opposite changes in gene expression for wild-type HOTAIR. In addition, in HCC cells, TGF-β1 has been shown to upregulate the expression of HOTAIR [48]. Silencing SMAD4 resulted in a lower HOTAIR expression, indicating that HOTAIR expression is regulated by TGF-β1 through SMAD4 activation.

CCL18 upregulates the expression of HOTAIR, and the knockdown of HOTAIR alleviates the CCL18-induced invasiveness of Esophageal squamous cell carcinoma (ESCC) cells. HOTAIR may also act as a competing endogenous RNA and become a sponge for miR-130a-5p, thus modulating the derepression of ZEB1 and promoting EMT in ESCC[49]. The transcription of the HOTAIR is controlled by the HNF4α-induced modulation of chromatin topology. In the mesenchymal-to-epithelial transition, HNF4α directly represses HOTAIR transcription. Mechanistically, HNF4α causes the release of a chromatin loop on HOTAIR regulatory elements, thus exhibiting an enhancer-blocking activity[50].

## HOTAIR as a driver of breast cancer

As a master regulator of cancer, HOTAIR is associated with various cellular and molecular mechanisms involved in carcinogenesis and cancer progression [51]. One of its targets is the c-Myc oncogene, which is frequently overexpressed in cancer. HOTAIR can repress c-Myc expression through several mechanisms [52]. One of which involves the recruitment of PRC2 to the c-Myc promoter region. It binds to the EZH2 subunit of PRC2, which then catalyzes the trimethylation of histone H3 at lysine 27 (H3K27me3) on the c-Myc promoter. This modification creates a repressive chromatin state that prevents transcriptional activation of c-Myc [53]. Another mechanism involves the direct binding of HOTAIR to the c-Myc protein through a specific RNA motif. This interaction inhibits the ability of c-Myc to bind to DNA and activate transcription[54]. In addition, HOTAIR can also modulate c-Myc expression through the regulation of microRNAs. HOTAIR has been shown to suppress the expression of several microRNAs that target c-Myc, leading to increased c-Myc expression [55].

Wu et al. [56] reported that HOTAIR facilitates BC progression via the promotion of EMT and metastasis by acting as a sponge between miR-129-5p and FZD7. This study observed that silencing HOTAIR preventes tumor growth in a xenograft model, while the silencing of miR-129-5p reversed HOTAIR gene silencing effects and FZD7 reinstated the inhibitory effect of miR-129-5p, suggesting that HOTAIR regulates the miR-129-5p/FZD7 axis. HOTAIR also promotes BC metastasis by controlling zinc finger E-box binding homeobox 1 (ZEB1)-dependent AKT signaling by targeting miR-601 via a sponging mechanism [57], highlighting its role as a potential target for BC therapy.

### Role of HOTAIR in the regulation of breast cancer metastasis

**Fig. 2:**
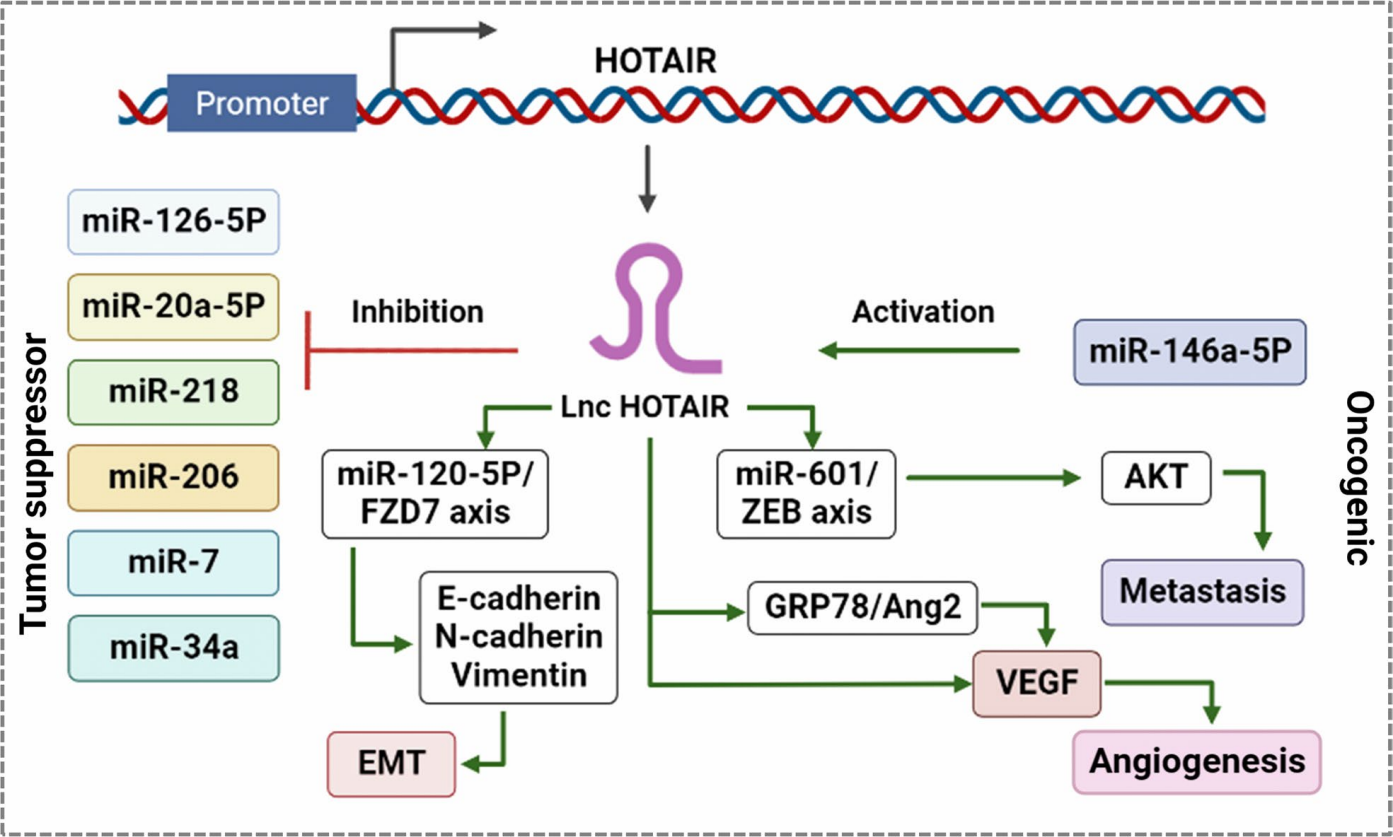
HOTAIR inhibits tumor suppressor miRNAs and activates pro-oncogenic miRNAs in BC. Activation of oncogenic miRNAs enhances the expression of EMT-specific proteins involved in metastasis by activating HOTAIR expression. HOTAIR regulates the miR-129-5p/FZD7 axis, which promotes EMT via the regulation of E-cadherin and N-cadherin vimentin. HOTAIR also activates the miR601/ZEB1 axis, which promotes metastasis by activating AKT. HOTAIR promotes angiogenesis by inducing the transcription of VEGF via direct interaction at the promoter or the activation of the GRP78/Ang2 axis

HOTAIR supports BC progression by controlling multiple signaling pathways as competitive endogenous RNA (Fig. 2). Its expression in primary BC is a strong indicator of metastasis. Aberrant HOTAIR expression promotes metastasis, drug resistance, and tumor recurrence in patients with various cancers [58]. Recent studies have reported that HOTAIR expression is upregulated in BC cells and tumor tissues, with its expression levels correlated with cell proliferation and metastasis. Furthermore, the silencing of HOTAIR induces apoptosis and inhibites cell proliferation. It promotes BC pathogenesis by linking miRNAs and the post-transcriptional network via physical interaction [59].

HOTAIR has also been shown to facilitate the BC cell proliferation by mediating miR-206 dependent BCL2L2 signaling [60]. This study found a positive association between HOTAIR and BCL2L2 levels in clinical BC samples, in particular, HOTAIR enhances BC cell proliferation by increasing BCL2L2 expression via the post-transcriptional regulation of miR-206 through sequestration. HOTAIR also contributes to tumor growth by negatively regulating miR-20a-5p [61]. A study by Liu et al. [62] reported that HOTAIR enhances BC cell invasion by facilitating the sulfation of chondroitin sulfate at N-acetyl galactosamine-4-sulfate moiety via the enhancement of the expression of chondroitin sulfotransferase.

In the early stages of BC, HOTAIR can promote cell growth and migration by modulating multiple signaling pathways related to EMT (Fig. 2), such as the FGFR and Wnt/catenin signaling pathways [63]. Induction of HOTAIR expression can facilitate the invasiveness and metastasis of BC cells by stimulating gene expression via the targeting of PRC2 and the alteration of the methylation of histone H3 lysine 27 [64]. Previous research has also found that HOTAIR can positively regulate the proliferation, migration and invasion of hormone receptor-positive BC cells and negatively control the cell cycle and apoptosis. The silencing of HOTAIR using siRNA markedly reduces the metastasis of BC cells by reducing Akt/JNK signaling and increase apoptosis by promoting p53 expression [65]. Collina et al. [66] assessed HOTAIR expression in tumor tissue from TNBC patients and found a strong association between higher expression level of HOTAIR and the metastasis of tumor cells to the lymph nodes and identified the involvement of androgen receptors in HOTAIR-dependent tumor progression.

Liang et al. [67] reported that TNBC tissue has higher expression levels of both HOTAIR and miR-146a-5p, which are associated with lower survival rates among TNBC patients. This study demonstrated that HOTAIR is positively regulated by miR-146a-5p using a TNBC cell line model and that both HOTAIR and miR-146a-5p enhance the metastasis of TNBC cells. However, the silencing of HOTAIR using siRNA mitigates the effect of miR-146a-5p.

In an analysis of the tumor samples from 348 TNBC patients using methylation-specific PCR, Lu et al. [68] observed a positive association between methylation at a downstream intergenic CpG island and its expression with clinical and pathological characters as well as the survival of patients. This study also found an association between the low risk of relapse and the high expression of HOTAIR using a multivariant Cox regression model. The study described the biological relevance of intergenic methylation on the regulation of the expression of HOTAIR in TNBC. Collectively, these studies suggest that the transcription of HOTAIR is controlled by epigenetic mechanisms, which leads to carcinogenesis.

Wang et al. [69] reported that exosomes containing HOTAIR in the serum correlate with BC progression. In particular, exosomal HOTAIR is positively associated with ErbB2 in tumor samples. Their study also validated the association between HOTAIR and ErbB2 using isogenic BC cell lines with and without the forced expression of ErbB2. Ren et al. [70] found that HOTAIR mediates the interaction of cancer-associated fibroblasts with BC cells during metastasis. This experiment demonstrated that TGF-β1 from CAFs promotes BC metastasis by inducing EMT via the promotion of HOTAIR expression. This study demonstrated that SMAD2/3/4 interacts with the promoter region of HOTAIR between − 386 and − 398 and between − 440 and − 452 to facilitate the transcription of HOTAIR. Using an orthotopic mouse model, this study proved that the silencing of HOTAIR provokes the lung metastasis of TNBC cells and tumor growth.

A study by Romero et al. [71] found that HOTAIR mediates hypoxia-induced metastasis of TNBC cells. It showed that hypoxia increases HOTAIR expression, but silencing HOTAIR using siRNA prevents the development of hypoxia-induced channel-like networks and branch points and increases miR-204 levels. Furthermore, the knockdown of HOTAIR alters the organization of the cytoskeleton by reducing the expression of FAK. These various studies highlight multiple routes through which HOTAIR can be targeted to suppress BC metastasis, thus it has the potential to be exploited as a target for BC treatment.

### Role of HOTAIR in resistance to breast cancer therapy

**Fig. 3:**
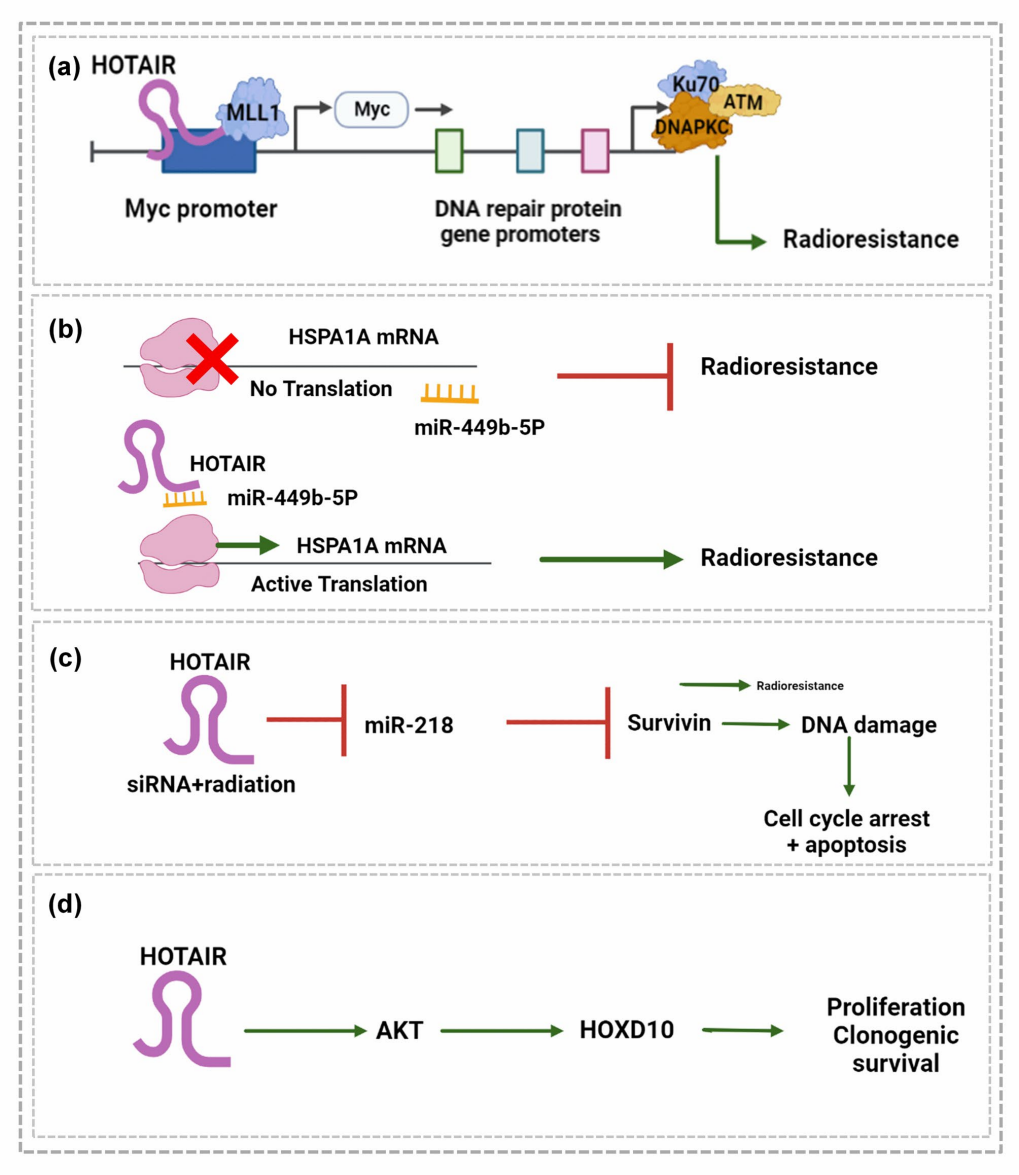
HOTAIR recruits EZH2 to the Myc promoter and facilitates the expression of Myc transcription factors. Myc binds to the gene promoter elements of DNA repair proteins Ku70/Ku80, DNA PKC, and ATM and promotes their expression to withstand the DNA damage caused by radiation in BC. b) miR 449b-5P suppresses radioresistance in BC by inhibiting the translation of HSPA1A. HOTAIR activates the translation and expression of HSPA1A by sponging miR-449b-5P. c) HOTAIR gene silencing and radiation therapy suppress miR-218, which in turn inhibits survivin, leading to DNA-damage dependent cell cycle arrest and apoptosis. d) HOTAIR activates AKP, which promotes proliferation and clonogenic survival via the activation of HOXD10

HOTAIR mediates resistance to radiation and chemotherapy in BC (Fig. 3). It has been reported that HOTAIR mediates the resistance of BC cells against radiotherapy by facilitating the binding of EZH2 to the promoter site of the Myc gene (Fig. 3a). As shown in Fig. 3a, HOTAIR induces radio-resistance by promoting the expression of DNA damage sensor proteins such as KU70, KU80, DNA-PKs, and ATM [72]. It also promotes radio-resistance in BC by enhancing the expression of HSPA1A through its role as a competing sponge. Figure 3b shows that functionally silencing HOTAIR enhances the radiosensitivity of BC cells by relieving the miR-449b-5p.

dependent repression of HSPA1A [73]. Hu et al. [74] also demonstrated that the silencing of HOTAIR using siRNA sensitizes BC cells to radiation therapy by inducing apoptosis via DNA damage and cell cycle arrest and by activating their associated pathways through the activation of miR-218 (Fig. 3c). Zhou et al. [75] demonstrated the impact of HOTAIR expression on radio-resistance in TNBC cells using a recombinant plasmid vector containing HOTAIR. This study revealed that HOTAIR increases the resistance to radiation therapy among TNBC cells by enhancing cell proliferation and clonogenic survival via the targeting of AKT pathway-dependent HOXD10, suggesting that HOTAIR suppression could improve therapeutic outcomes.

**Fig. 4:**
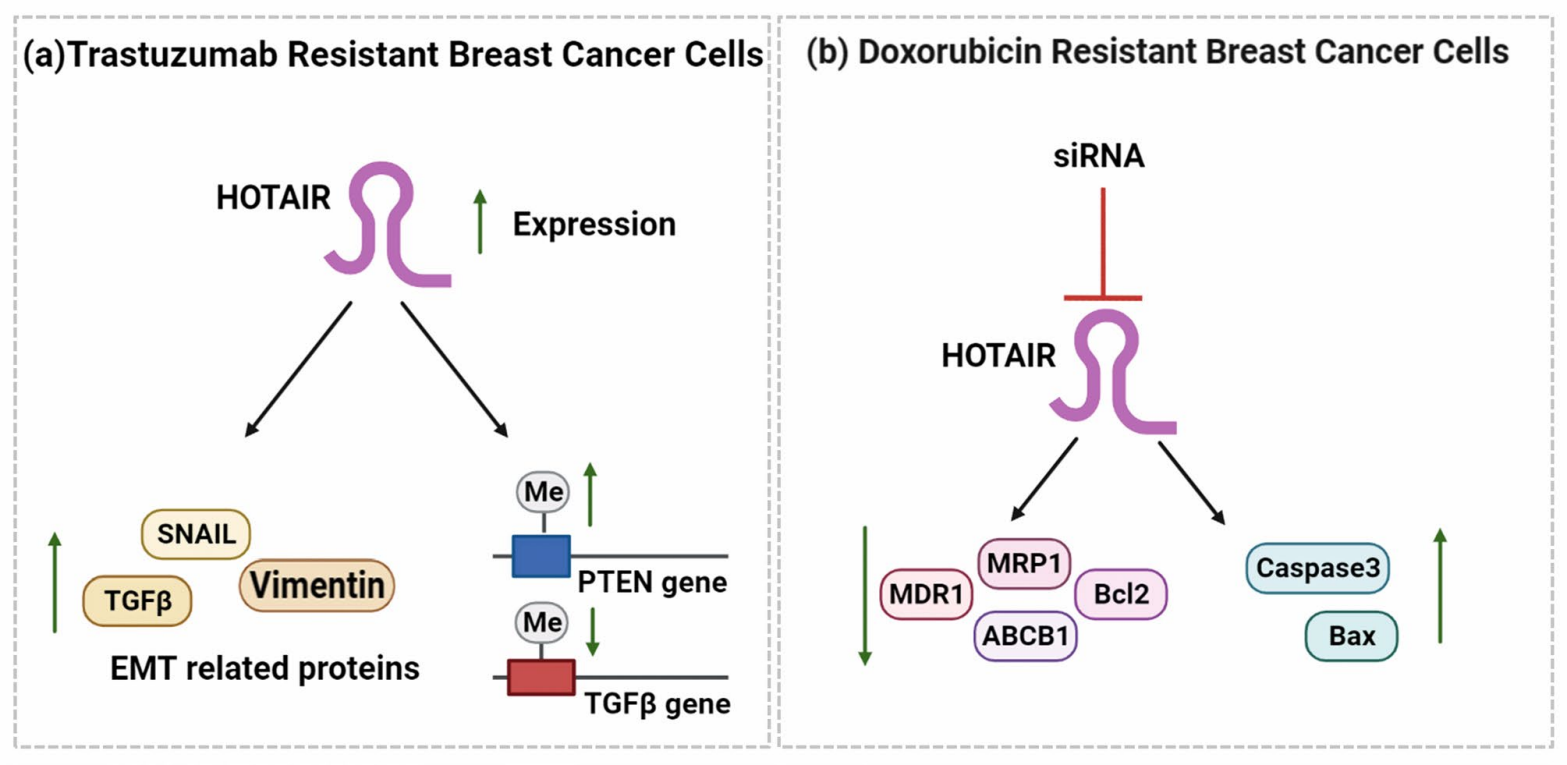
HOTAIR is overexpressed in trastuzumab-resistant BC cells and upregulates the expression of EMT-related proteins SNAIL, TGFß, and vimentin. At the same time, it also upregulates PTEN gene promoter methylation and downregulates TGFß gene promoter methylation. The siRNA-mediated silencing of HOTAIR in DOX-resistant BC cells downregulates the expression of oncogenic and anti-apoptotic proteins MDR1, MRP1, ABCB1, and Bcl2 and upregulates the pro-apoptotic proteins caspase 3 and Bax

A study on the clinical treatment of BC patients with E2 observed that HOTAIR can mediate endocrine therapy resistance via the hub gene ESR1 by targeting miR-130b-3p in BC patients [76]. It can also mediate trastuzumab resistance in BC cell lines by upregulating TGF-β, vimentin and Snail expression, downregulating E-cadherin levels, and increasing the methylation of PTEN gene (Fig. 4a). However, the silencing of HOTAIR by siRNA sensitizes BC cells to trastuzumab by reducing the expression of TGF-β, vimentin and Snail and reduces the BC growth in a mouse model [77].

HOTAIR also mediates Doxorubicin (DOX) resistance in BC cells (Fig. 4b). The silencing of HOTAIR with siRNA sensitizes DOX-resistant BC cells by reducing proliferation, inducing apoptosis, and impeding the activity of PI3K/AKT/mTOR signaling. In addition, lower Bcl2 and caspase 3, higher BAX, and lower MDR1, MRP1, and ABCB1 expression levels have been observed in HOTAIR-silenced DOX-resistant BC cells, illustrating the significance of HOTAIR in the induction of DOX resistance in BC [78].

HOTAIR promotes the drug and radio-resistance of BC by inducing EMT and stemness. EMT is one of the key mechanisms of cancer progression and is required for the maintenance of cancer stem cells (CSCs). TGF-β1-induced HOTAIR enhances the ability of BC cells to form colonies by promoting EMT via the increase in the expression of vimentin, a mesenchymal marker, and the reduction of E-cadherin, an epithelial marker [79]. In BC, HOTAIR can maintain EMT and CSCs by suppressing miR-7 via c-myc and TWIST expression [80]. HOTAIR can also regulate autophagy, which promotes BC progression and stem cell properties via the direct interaction with autophagy-related and invasion-associated genes such as MMPs and β-catenin or the indirect interaction with miR-34a [29]. HOTAIR can also regulate stemness in BC by upregulating the expression of the stemness-related genes CD44, STAT3, and ALDH2 [81]. BC cells enriched with CSCs also exhibit higher expression of HOTAIR compared to their normal counterparts, while the modulation of HOTAIR expression using full-length HOTAIR enhances the self-renewal capacity of CSCs by increasing SOX2 expression through miR-34a [82]. This study also reported that HOTAIR can control the transcription of p53 in CSCs by indirectly regulating p21.

Overall, the sponging mechanisms of HOTAIR associated with the regulation of gene expression in BC involve sequestering miRNAs to prevent their normal function, resulting in the higher expression of target genes. HOTAIR does this by sponging mRNA-interfering complementary RNA (miRNA) molecules that would normally target and degrade the mRNA of downstream genes. HOTAIR contains miRNA response elements that can bind to miRNAs, sequestering them from their natural targets and reducing their ability to suppress gene expression. By binding miRNAs, HOTAIR effectively competes with endogenous transcripts that contain same miRNA response elements, leading to an increase in the expression of the target genes. HOTAIR’s sponging mechanisms have been shown to regulate the expression of multiple genes involved in BC, including HER2, BRCA1, and PTEN. Hence, HOTAIR inhibition represents a promising strategy for improving responses to therapeutic regimens in treatment-refractory patients.

## The role of HOTAIR in breast cancer management

### Use of HOTAIR in assessing the breast cancer risk

Single nucleotide polymorphisms (SNPs) are reported to upregulate HOTAIR expression and are correlated with an increased risk of BC. In addition, the GG/AG genotype of rs720778 and GA genotype of rs4759314 are reported to be associated with lower disease-free survival (DFS) and overall survival (OS) and DFS, respectively, in some populations of BC patients [83]. A similar study in India by Rajagopal et al. [84] reported that a combination of SNPs in NME1 (rs16949649 T > C and rs2302254 C > T) and HOTAIR (e.g., rs920778 C > T and rs1899663 G > T) increased the risk of BC in premenopausal women carrying mutant alleles. Another population-based case-control study by Yan et al. [85] in China reported that rs920778 in HOTAIR significantly enhances the risk of BC and is associated with age at menopause, the number of abortions, and family history and significantly enhanced the risk of BC. Another study in Turkish women using both codominant and recessive inheritance models reported that rs920778 (T-C) in the enhancer region of HOTAIR enhances the risk of BC. This study demonstrated that rs920778 is associated with the clinicopathological characteristics of breast tumors, such as their histological grade, tumor size, and metastatic ability [86]. Li et al. [87] also clarified the relationship between susceptibility to BC and SNPs in HOTAIR based on the meta analysis of 5322 patient samples from 11 studies. Their study predicted a strong association between rs4759314 and BC risk using codominant, dominant, and recessive models. A population-based study from Iran reported that SNP rs920778 HOTAIR has a positive correlation with the risk of BC, while rs12826786 and rs1899663 have an inverse correlation [88]. This study also demonstrated that TGAC, CTAT, and TTAT polymorphisms substantially lower the BC risk. In contrast, Mathias et al. [89] predicted no correlation between BC susceptibility and rs4759314, rs920778, rs12826786, and rs1899663 in HOTAIR.

**Table 1:** Summary of SNPs and the risk of breast cancer

SNP	Genotype	Risk	Ref
rs720778	GG/AG	Leads to lowest disease-free survival	[83]
rs4759314	GA	Reduces overall survival	
rs920778	C > T		
rs1899663	G > T	Increases the risk of BC in premenopausal women	[84]
rs920778	C > T	Enhances the risk of BC	[85]
rs920778	T-C	Enhances the risk of BC associate with histological grade, tumor size, and distinct metastatic ability.	[86]
rs4759314	AG/AA	Increases BC risk.	[87]
rs920778	T > C	Progressive correlation with risk	[88]
rs1899663	G > T	Negative correlation with risk.	
rs1899663 rs4759314	-	No correlation with the risk of BC.	[89]

### Use of HOTAIR in the diagnosis and prognosis of breast cancer

Various studies have confirmed the potential of HOTAIR as an informative diagnostic and prognostic tool for the detection and management of BC. In particular, the aberrant expression of HOTAIR has been reported for different BC subtypes [90], thus numerous studies have evaluated the efficiency of serum HOTAIR as a biomarker for the diagnosis and prognosis of BC in patients using various models. Serum HOTAIR levels are reported to be higher in BC patients than in healthy individuals with higher discriminating power [91]. HOTAIR has also been identified as a prognostic marker of BC using univariate Cox regression analysis of differentially expressed lncRNAs in normal breast tissue and BC samples [92]. This study predicted a correlation between OS and HOTAIR levels in BC tissue. Another study evaluated the serum levels of a HOTAIR SNP (rs12826786) and Nestin as screening markers of BC by genotyping HOTAIR gene variants using PCR-RFLP analysis [93]. This study reported a significant difference in HOTAIR gene alleles between normal healthy individuals and BC patients, especially T/T genotypes, which are more common among BC patients, and C/C genotypes, which are lower in normal healthy individuals.

Profiling experiments have also been used to evaluate the diagnostic potential of HOTAIR during different stages of BC [94]. This study demonstrated that HOTAIR levels are considerably higher in stage II, III, and IV tumor tissue samples than in stage I samples. The expression profile of HOTAIR is also notably higher in stages III and IV than in stage II, indicating the potential in using HOTAIR to target BC metastasis. Using co-expression network analysis, Li et al. [95] identified HOTAIR as one of the four lncRNAs associated with BC prognosis and developed a corresponding prognostic model. Using this model, a risk-adaptive management strategy for BC was identified for accurate risk assessment and the selection of optimal strategies, thus providing novel insights for clinical applications in BC management. Furthermore, a meta-analysis of differentially expressed HOTAIR in 2192 BC samples from 21 studies revealed a notable association between HOTAIR expression and OS and DFS, suggesting that HOTAIR is a reliable marker for patients with TNBC [96]. predicted a correlation between the expression of HOTAIR and the worst prognosis using the high-throughput sequencing of cohorts and TCGA validation analysis. Their study also highlighted the oncogenic potential of HOTAIR using RNA interference technology.

The silencing of HOTAIR using siRNA reduces the proliferation of BC cells by promoting programmed cell death and inducing cell cycle arrest at the Go/G stage [97]. Further, the response of circulatory HOTAIR to neoadjuvant chemotherapy (NAC) predicts the correlation with DFS in BC patients by quantifying HOTAIR in serum using quantitative real-time PCR [98]. Using Kaplan-Meier curves and log-rank tests, this study predicted that BC patients with elevated levels of serum HOTAIR exhibit a poor clinical response to NAC and the lowest DFS, indicating that serum HOTAIR is a potential predictor of NAC in BC patients. Another study by Zhang et al. [99] described the biomarker potential of serum HOTAIR in BC patients using PCR-based detection. This study measured the DNA of HOTAIR in BC patients and healthy controls in the discovery and independent-validation stages and found that HOTAIR is present in the serum as DNA fragments rather than RNA. Moreover, the diagnostic potential of serum HOTAIR has been predicted using the receiver operating curve and area under the curve metrics, with higher levels of HOTAIR DNA detected in the serum compared to healthy controls [99]. This study also found that the cutoff value of relative concentration for HOTAIR DNA for the diagnosis of BC was 0.30, with a sensitivity and specificity of 80.0% and of 68.3%, respectively, suggesting that HOTAIR DNA is a potential biomarker for the diagnosis of BC.

In another study, Zhang et al. [100] estimated the HOTAIR levels in the plasma of BC patients before and after surgery and compared them with the plasma levels of CA153 and CEA for BC diagnosis. Their study found that HOTAIR levels are strongly related to lymph node metastasis. However, after surgery, the level of plasma HOTAIR are significantly lower and have a moderate relationship with HOTAIR expression. Furthermore, the diagnostic potential of plasma HOTAIR has been confirmed using receiver operating curves and logistic regression with significantly higher diagnostic power and specificity over CA153 and CEA. The diagnostic power of plasma HOTAIR was further enhanced by combination with plasma CA153 and CEA in BC patients [100]. Ezzatizadeh et al. [101] reported that radiation therapy has a varying prognostic influence on HOTAIR levels in the peripheral blood of BC patients, indicating its potential as a blood marker for the diagnosis of BC [101]. Insitu hybridization (ISH) analysis of BC tissue microarrays by Evans et al. [102] confirmed the association between HOTAIR and the recurrence of TNBC. HOTAIR mRNA levels were significantly higher in BC samples with high recurrence scores, while mRNA levels were low in BC samples with low recurrence scores, indicating that HOTAIR has the potential for use as a recurrence marker of BC [102].

### HOTAIR in breast cancer therapy

Experimental studies have found that HOTAIR is a suitable therapeutic target for BC, with the mechanisms reported for the inhibition of HOTAIR expression listed in Table 1. Delphinidin (DEL), a major anthocyanidin present in pigmented fruits and vegetables, has been found to inhibit 1-methyl-1-nitrosourea-induced BC in rats [103] via the upregulation of miR-34a by targeting the expression of HOTAIR. The inhibition of HOTAIR by DEL is coupled with the reduced occupancy of EZH2 and histone H3 Lys27 trimethylation at the promoter site of miR-34a in TNBC cells. This study also demonstrated that the upregulation of HOTAIR impedes the effect DEL on miR-34a and Wnt/catenin signaling in TNBC cells. Furthermore, a glucoside derivative of DEL (DEL-3-glucoside) inhibits tobacco-specific nitrosamine- and benzopyrene-induced carcinogenesis in normal breast epithelial cells by suppressing the expression of HOTAIR [104]. Further, in a xenograft model of TNBC, DEL-3-glucoside reduces the expression of HOTAIR and AKT by promoting the expression and binding of IRF1 to the HOTAIR promoter. Chen et al. [105] also reported that calycosin and genistein treatment reduces HOTAIR expression by restricting Akt phosphorylation in MCF-7 BC cells.

Calycosin inhibits BC cell proliferation and induces apoptosis in both ER-positive and ER-negative BC cell lines. The anti-tumor effect of calycosin is promoted by the knockdown of lncRNA HOTAIR and attenuated by its overexpression. The downregulation of lncRNA HOTAIR by calycosin leads to the reduced expression of HuR and IGF2BP1, while the upregulation of HuR and IGF2BP1 due to lncRNA HOTAIR overexpression is weakened by calycosin. The inhibited growth of BC cells due to calycosin is attributed to the downregulation of HuR and IGF2BP1 through the suppression of lncRNA HOTAIR. The binding of HuR and IGF2BP1 to lncRNA HOTAIR has been detected, suggesting an interaction between these two proteins and lncRNA HOTAIR [106].

Ozes et al. [107] reported that tumor-specific peptide nucleic acid (PNA3), which is complementary to the 89-mer domain, reduced the invasion of BC cells by increasing the sensitivity to chemotherapy via the reduction of the NF-kB-dependent expression of MMP9 and IL-6. Wang et al. [108] clinically validated the treatment of TNBC cells with a combination of imatinib and lapatinib, which synergistically reduce the expression of HOTAIR by regulating β-catenin recruitment at the promoter via interference with the LEF1/TCF4-binding site. This was experimentally proven by the chromatin immunoprecipitation of β-catenin along with the genomic region containing the TCF4/LAF1 site in TNBC cells treated with imatinib and lapatinib. In addition, the small-molecule AC1Q3QWB (AQB) has been shown to inhibit BC growth [109]. This study observed that AQB enhances the expression of tumor suppressors by interfering with the recruitment of PRC2 via the disruption to the interaction of HOTAIR with EZH2. In addition, AQB treatment upregulated HOTAIR target genes such as APC2 via suppressing Wnt/catenin signaling and enhancing the transcription by RNA pol II. Another small compound, AC1NOD4Q (ADQ), has been reported to inhibit BC metastasis by blocking HOTAIR activity through interference with EZH2 interaction [110]. Blocking HOTAIR activity can also impair H3K27-dependent trimethylation of the target gene and subsequent inhibition of the Wnt/catenin pathway in an orthotopic BC model. Using RIP and EMSA, it was confirmed that AQD prevents the interaction of HOTAIR with EZH2 by binding to a specific domain of HOTAIR.

Metformin (MET), an anti-diabetic drug, is widely used for the treatment of various cancers, including BC. In TNBC cells, it inhibits HOTAIR expression by affecting the methylation pattern of CpG islands in the promoter region and reverses EMT by lowering the vimentin and β-catenin levels. This treatment also reduces the invasiveness and migratory potential of TNBC cells in transwell and scratch assays [111]. Furthermore, CCT137690, which is a small-molecule inhibitor of aurora kinase (AURK), inhibits the expansion of TNBC cells via the reduction of HOTAIR levels [112] by affecting AURK and its network proteins [113].

HOTAIR expression is also successfully inhibited by natural compounds. For example, the natural steroidal saponin dioscin inhibits stem-like properties and EMT in BC [114] and HOTAIR expression in gastric cancer [115]. Other studies have reported that polyphyllin I, a small molecule from *Paris polyphylla*, can disrupt HOTAIR interaction with its upstream regulators and downstream mediators [116] and inhibit the cell cycle and induce apoptosis in BC [117]. In addition, berberine, a natural alkaloid inhibitor of BC [118], and gefitinib inhibit HOTAIR expression by inducing miR-34a-5p expression in lung cancer cells [119]. Collectively, these studies indicate that the modulation of HOTAIR expression by natural compounds, small molecules, or anticancer inhibitors significantly reduces BC progression to metastasis in clinical settings. Hence, targeting HOTAIR could improve current therapeutic regimens by disrupting resistance mechanisms.

**Table 1:** Inhibition of HOTAIR expression by bioactive compounds, anticancer drugs and HOTAIR inhibitor

Bioactive compounds/ anticancer drugs/HOTAIR inhibitor	Mechanism of action	Ref
Delphinidin (DEL)	• Inhibits 1-methyl-1-nitrosourea-induced BC in rats by upregulating miR-34a via the targeting of HOTAIR expression • Reduces the occupancy of EZH2 and histone H3 Lys27 trimethylation at the promoter site of miR-34a	[103]
DEL-3-glucoside	• Inhibits nitrosamine and benzopyrene-induced carcinogenesis in normal breast epithelial cells by reducing the expression of HOTAIR. • Reduces the expression of HOTAIR and Akt by increasing the expression and binding of IRF1 to the of HOTAIR promoter	[104]
Calycosin and genistein	• Reduces HOTAIR expression by reducing Akt phosphorylation in MCF-7 BC cells	[105]
Peptide nucleic acid (PNA3)	• Reduces the invasion of BC cells by increasing the sensitivity to chemotherapy via reduction in NF-kB dependent expression of MMP9 and IL-6	[107]
Imatinib and lapatinib	• Synergistically reduces the expression of HOTAIR by regulating β-catenin recruitment at the promoter via interference with the LEF1/TCF4-binding site	[108]
AC1Q3QWB (AQB)	• Enhances the expression of tumor suppressors by interfering with the recruitment of PRC2 via the disruption of the interaction between HOTAIR and EZH2 • Upregulates HOTAIR target genes especially APC2 via the suppression of Wnt/catenin signaling and the promotion of transcription via RNA pol II	[109]
AC1NOD4Q (ADQ	• Inhibits BC metastasis by blocking HOTAIR activity via interference with the EZH2 interaction • Impairs the H3K27-dependent trimethylation of the target gene and subsequent inhibition of the Wnt/catenin pathway in an orthotopic BC model • Prevents the interaction of HOTAIR with EZH2 by binding to a specific HOTAIR domain	[110]
Metformin (MET)	• Inhibits HOTAIR expression by affecting the methylation pattern of CpG islands in the promoter region and reverses EMT by lowering vimentin and β-catenin levels • Reduces the invasiveness and migratory potential of TNBC cells	[111]
Aurora kinase inhibitor (CCT137690)	• Inhibits the expansion of TNBC cells by reducing HOTAIR levels via its influence on AURK and associated network proteins	[112] [113]
Steroidal saponin (Dioscin)	• Inhibits stem-like properties and EMT in BC.	[114]
	• Reduces HOTAIR expression in gastric cancer	[115]
Polyphylla I	• Disrupts the interaction of HOTAIR with its upstream regulators and downstream mediators.	[116]
	• Inhibits the cell cycle and induces apoptosis in BC	[117]
Berberine	• Inhibits BC and gefitinib and inhibits HOTAIR expression by inducing miR-34a-5p expression in lung cancer cells	[118] [119]

HOTAIR has been reported to be an upstream regulator of MALAT1, and promising results have been obtained from the use of MALAT1 and HOTAIR in regulating oncogenic immune-modulatory proteins CD80 and MSLN in tumor-associated macrophages (TAMs) [120]. This study also investigated the association between HOTAIR and MALAT1 as regulators, highlighting the regulatory role of MALAT1 and HOTAIR lncRNAs in controlling the tumorigenic activity of TAMs in BC and examining the impact of manipulating MALAT1 and HOTAIR on the expression of CD80 and MSLN in TAMs for HER2 + and TNBC BC. Another study has revealed a significant difference in HOTAIR expression between BC and normal tissue and significant associations between HOTAIR gene expression and the tumor size and margin [121]. However, there is no significant difference in the expression of HOTAIR lncRNA in MCF7 and MDA-MB-231 cancer cell lines compared with normal MCF-10A cells. This study also reported that circulating HOTAIR has high sensitivity and specificity as a diagnostic marker for BC, with a significant correlation between HOTAIR expression and OS.

The present review offered a new perspective on the post-transcriptional and epigenetic regulation of HOTAIR expression, highlighting the role of microRNAs and RNA-binding proteins. Moreover, we provide novel insights into the potential targeting of HOTAIR as a therapeutic strategy for overcoming drug resistance and inhibiting metastasis in BC. Our review also addresses the current limitations in the understanding of HOTAIR and offers a critical analysis of the existing literature, thus providing a basis for future research in this field. Overall, this review provides a unique perspective of HOTAIR and its potential as a therapeutic target for BC management.

This review explored the upstream and downstream targets of HOTAIR and how it interacts with other molecular pathways involved in BC progression. For instance, HOTAIR has been shown to promote BC progression by regulating the miR-129-5p/FZD7 axis, where HOTAIR acting as a sponge for miR-129-5p, while FZD7 is a target of miR-129-5p. *In vivo* analysis has demonstrated that the knockdown of HOTAIR can inhibit tumor growth [56]. HOTAIR also serves as a competing endogenous RNA in the newly discovered regulatory axis involving HOTAIR, miR-203, and CAV1, highlighting a potential new target for the development of BC therapies[122]. HOTAIR also sequesters miR-130a-3p and promotes Suv39H1-mediated AKT/mTOR signaling, which facilitates BC cell growth and metastasis, with the effects of HOTAIR knockdown on BC abolished by restoring Suv39H1 expression [123].

One of the insights that can be drawn from this review of HOTAIR is its role in the regulation of BC metastasis via the regulation of the genes involved in cell invasion and migration. This review also highlights the involvement of HOTAIR in drug resistance, which is a significant obstacle in cancer treatment. It confers resistance to chemotherapy and targeted therapies by regulating the genes that control drug metabolism, DNA repair, and apoptosis, highlighting the potential of targeting HOTAIR as a strategy to overcome drug resistance in cancer. Another important insight is that HOTAIR has the potential as a prognostic marker and a therapeutic target for BC treatment. For example, its overexpression has been linked to poor BC prognosis, where it is associated with lymph node metastasis, advanced tumor stages, and lower OS, and to liver metastasis, where it is linked to tumor recurrence and lower survival. However, further research is required to develop effective and safe HOTAIR-targeting therapies. Overall, the potential of HOTAIR as a metastatic, drug-resistant, and prognostic regulator highlights the importance of understanding the molecular mechanisms underlying cancer progression and identifying novel therapeutic targets for cancer treatment.

## Conclusion

In conclusion, HOTAIR is a long non-coding RNA that has been identified as a key regulator of BC metastasis, drug resistance, and prognosis. Its upregulation has been associated with the development of aggressive BC phenotypes, such as TNBC, and poor patient outcomes. Mechanistically, HOTAIR has been shown to promote metastasis by regulating EMT and enhancing cancer cell invasion and migration. It also contributes to drug resistance by regulating the genes involved in drug metabolism, such as P-glycoprotein. In addition, HOTAIR has been proposed as a potential prognostic biomarker and therapeutic target for BC.

## Future directions

Research on HOTAIR and its role in BC is rapidly evolving, and promising directions for future research include the elucidation of HOTAIR’s regulatory network, the development of HOTAIR-targeting therapies, and clinical validation of HOTAIR as a prognostic biomarker. Although HOTAIR has been shown to regulate several genes and signaling pathways, its full regulatory network is not yet fully understood. HOTAIR has been proposed as a potential therapeutic target for BC, and several approaches targeting HOTAIR expression have been explored. Future studies could focus on the development of more efficient and specific HOTAIR-targeting therapies, such as small-molecule inhibitors or gene therapy approaches. While several studies have identified HOTAIR as a potential prognostic biomarker for BC, its clinical utility has yet to be validated in large-scale clinical trials. Future studies could focus on validating HOTAIR as a prognostic biomarker, potentially leading to improved patient stratification and treatment decision-making.

## Data Availability

Not applicable.
